# A Refinement of Recurrence Analysis to Determine the Time Delay of Causality in Presence of External Perturbations

**DOI:** 10.3390/e22080865

**Published:** 2020-08-06

**Authors:** Emmanuele Peluso, Teddy Craciunescu, Andrea Murari

**Affiliations:** 1 Department of Industrial Engineering, University of Rome “Tor Vergata”, via del Politecnico 1, 00133 Roma, Italy; 2 National Institute for Laser, Plasma and Radiation Physics, RO-077125 Magurele-Bucharest, Romania; c.teddy@ifa-mg.ro; 3 Consorzio RFX (CNR, ENEA, INFN, Universita di Padova, Acciaierie Venete SpA), Corso Stati Uniti 4, 35127 Padova, Italy; andrea.murari@euro-fusion.org

**Keywords:** joint recurrence plot, conditional transfer entropy, synchronization, autoregressive models, causality in time series

## Abstract

This article describes a refinement of recurrence analysis to determine the delay in the causal influence between a driver and a target, in the presence of additional perturbations affecting the time series of the response observable. The methodology is based on the definition of a new type of recurrence plots, the Conditional Joint Recurrence plot. The potential of the proposed approach resides in the great flexibility of recurrence plots themselves, which allows extending the technique to more than three quantities. Autoregressive time series, both linear and nonlinear, with different couplings and percentage of additive Gaussian noise have been investigated in detail, with and without outliers. The approach has also been applied to the case of synthetic periodic signals, representing realistic situations of synchronization experiments in thermonuclear fusion. The results obtained have been very positive; the proposed Conditional Joint Recurrence plots have always managed to identify the right interval of the causal influences and are very competitive with alternative techniques such as the Conditional Transfer Entropy.

## 1. Multiple Causality between Time Series

Most specialists agree to trace the concept of synchronization back to Huygens and his study on what we define today as antiphase synchronized pendula. Presently, the study of synchronization between dynamical systems is generally focused on determining the nature of the coupling among the observables involved. As different forms of mutual influence exist, different synchronization typologies can be defined as well. Phase synchronization, generalized synchronization, and lag synchronization are just some examples [1]. Several valuable theoretical approaches have been proposed, see, e.g., in [2,3,4], for their assessment. In practical applications, the evolution of the generalized and phase synchronizations between two quantities are among the most widely studied, while lag synchronization is often aimed at inferring the delay of the maximal influence between two observables.

As dynamical systems can be observed in all of natural science, from medicine and biology to engineering and physics, the interest on their coupling, dynamic, and relation is a still very active topic of research. In biology, for example, Pavlova et al. [5] applied the detrended fluctuation analysis (DFA) to characterize complex interactions between neurophysiological signals, to avoid destroying the long-range correlation in the original data using prefiltering techniques. In medicine, studies about brain signals have motivated the development of different new approaches such as the use of the complex networks to understand the relationship between brain organization and behavior [6]. Considering climate application also, a new method, called the Reservoir Computing Causality method (RCC), has been recently developed by Huang et al. [7], and also compared with the Extended Convergent Cross Mapping (ECCM) [8] to identify the causal direction, coupling delay between two quantities described by two time series. This method has the advantage of not requiring the estimation of the embedding dimension and of the delay time. In engineering, Tirabassi et al. studied the relation between inferred functional networks and the underlying structural networks between two dynamical systems, a simulated set of Kuramoto oscillators, and an experimental set of Rössler chaotic electronic circuits, finding that there were regimes of the dynamics in which the functional connectivity of the system mimics the structural one, or in which the former is a good approximation of the latter [9]. Zhao et al. then showed the reciprocal characterization of time series and multilayer networks and introduced the concept of interlayer entropy between two quantities aimed at measuring the strength of interrelationships from one layer to another, demonstrating that in networks it is the equivalent of transfer entropy in time series [10]. In telecommunication and electronics, integrated circuits and their design [11], both due to the wide range of dynamics that can be explored and to the huge amount of complex data that can be produced, represent an interesting field of research for many applied oriented studies like chaos-based secure communications [12] using the so called snap circuits.

The investigation of the causal influence of dynamic systems is therefore a very difficult problem, far from being completely solved [13,14] even for two time series. In addition, as complex systems can very rarely be considered isolated and immune from external influences, it might be necessary to consider additional influences that might affect our understanding and lead, in practical applications, to wrong conclusions about the physical mechanisms behind the measurements acquired, especially when interested at the time delay of causality. This article is a contribution to this line of work, providing a new technique for the analysis of the synchronization between two quantities to assess the time delay of causality in presence of external perturbations. The methodology can be easily generalized to more quantities, as shown for a case study in Section 6.

The proposed approach is based on the recurrence plots [15]. Results have been compared with the Transfer Entropy and its extension, the so-called Conditional Transfer Entropy [16].

Section 2 recalls the Recurrence Plots and defines the new Conditional Joint Recurrence Plot (CJRP), while Section 3 reviews the Transfer Entropy and its extension the Conditional Transfer Entropy. Section 4 is consequently dedicated to the description of the tests performed on an autoregressive system, while Section 5 shows the results obtained for a case study dealing with periodic synthetic functions aimed at mimicking realistic situations of synchronization experiments in thermonuclear fusion. Section 6 shows an example of the application of the CJRP to four autoregressive quantities, to illustrate the actual potential of the approach to be extended to more complex situations. Section 7 covers the issue of reliability in the presence of outliers in the data, while the future planned applications of the tool used herein are discussed in Section 8. The last section draws the main conclusion of the work.

## 2. Refinement of Joint Recurrent Plots

A RP is actually a plot of a matrix, showing the occurrences in times at which a phase space trajectory visits the same area in phase space [15]. Mathematically it is described by a recurrence matrix:

(1) $$RP_{ij}=\Theta \left(\epsilon -\|y_{i}-y_{j}\|\right),\;y_{i,j}\in R^{m},\;i,j\in 0,N$$

where *N* is the number of samples, “*m*” the dimension of the embedded phase space, ‖°‖ is a norm, and ϵ is a threshold, usually around 10% of the average or of the maximal or mean diameter of the attractor [15]. RP can be extended with the notion of the joint recurrence plots (JRP), taking the Hadamard product of two RPs and so allowing investigation of the possible simultaneous occurrences between two quantities or two dynamical systems. Mathematically, in the case of two time series [15]

(2) $$JRP_{ij}^{xy}=\Theta \left(\epsilon_{x}-\|x_{i}-x_{j}\|\right)\Theta \left(\epsilon_{y}-\|y_{i}-y_{j}\|\right),\;y_{i,j}\in R^{m},\;x_{i,j}\in R^{n},\;i,j\in 0,N$$

With an analogous meaning for the symbols used. From Equation (2), it emerges clearly that it is easy to include further quantities in the analysis [15], by simply performing the Hadamard product (“⊙”) of the JRP with a RP:

(3) $$JRP_{xyz}={\mathrm{JRP}}_{\mathrm{xy}}⊙{\mathrm{RP}}_{z}$$

Various variants have been introduced for the RR and JRP [15]. Here, we propose the new conditional joint recurrence plot as

(4) $$CJRP_{xy|z}={\mathrm{JRP}}_{\mathrm{xy}}⊙\left(1-{\mathrm{JRP}}_{\mathrm{zy}}\right)$$

While (3) allows estimating the same occurrence in phase space among three quantities, without distinguishing the actual synchronization between them, (4) has been conceived to filter out the influence of “*z*” on “*y*”, to better investigate the one between “*x*” and “*y*”.

The idea behind the methodology developed for the application of (4) is as follows. A key parameter of the RP and of their extensions is the threshold ϵ in (1). The higher its value, the more elements equal 1 in the matrix; the lower its value, the more difficult it is to get a “1”. This reflects the fact that, when reducing the threshold, the closer the points on the attractors have to be in order to count as recurrences. As the aim is determining the delay between a driver “*x*” and response observable “*y*” in presence on external perturbation “*z*”, to exclude the effect of unwanted occurrences, one can proceed by fixing the threshold $\epsilon_{JRP_{xy}}$ around its optimal value and iteratively reducing the threshold $\epsilon_{JRP_{yz}}$. Then, for each value of $\epsilon_{JRP_{yz}}$, a scan in the delay between the two signals is performed, to determine the time of maximum interaction between the two systems.

Reducing progressively $\epsilon_{JRP_{yz}}$ in Equation (4), starting from a value $\epsilon_{JRP_{xy}}\sim \epsilon_{JRP_{yz}}$, the number of occurrences between *z* and *y* decreases. Only the points on the attractors of *z* that are close to those of *y* cause the Heaviside function to output a “1” and consequently a “0” in the matrix of $CJRP_{xy|z}$. In this way, the occurrences between *x* and *y* are preserved and the ones between *z* and *y* are progressively sifted out. Scanning the value of $\epsilon_{JRP_{yz}}$, the CJRP reaches a stable region, in which it is possible to extract reliably the desired information with specific indicators (see later). On the contrary, decreasing the threshold $\epsilon_{RP_{z}}$ in Equation (3), the resulting JRP provides the occurrences where both *x* and *z* strongly influence *y*, but the relative importance of the two cannot be determined. To conclude, the direct use of the $JRP_{xy}$ would not be able to single out spurious occurrences induced from external perturbations on the response system.

Besides allowing a synthetic visualization of complex processes recurrences, RPs, JRPs, and CJRPs may be used also to derive important properties of a system phase space. It has to be considered in fact that single isolated points correspond to infrequent states with a short persistence, while the existence of vertical and horizontal lines accounts for states relatively stable in time. The most interesting features are the diagonal lines, corresponding to trajectories in the phase space visiting the same region at different times. Short diagonals are characteristic for weakly correlated, stochastic, or chaotic processes, while long diagonals occur for deterministic processes [15]. Thus, recurrence quantification analysis [17] provides a number of useful estimators, which are derived from the visual features occurring in RPs. In the following, three main estimators are considered [15,17]: the mean diagonal length (L), the determinism (DET), and the recurrence rate (RR). L and DET are related to the periodic behavior of the system analyzed, while RR estimates the density of recurrences. For coupled systems, the length of a diagonal is proportional to the duration of the local evolution of the trajectories; the determinism, defined as the percentage of recurrence points forming the diagonal lines and consequently related to the length of the diagonal lines, allows measuring the predictability (or determinism) of the system. Finally, the recurrence rate represents an indicator for correlated recurrences.

A detailed description of these estimators can be found in the literature [15,17] and is out of the scope of this article. In Equations (5)–(7), the mathematical expressions for L, DET, and RR are provided according to [15,17]

(5) $$L=\frac{\sum_{l=l_{min}}^{N}\;\;lP\left(l\right)}{\sum_{l=l_{min}}^{N}\;\;P\left(l\right)}$$

(6) $$DET=\frac{\sum_{l=l_{min}}^{N}\;\;lP\left(l\right)}{\sum_{l=1}^{N}\;\;lP\left(l\right)}$$

(7) $$RR=\frac{1}{N^{2}}\sum_{i,j=1}^{N}RP_{ij}$$

where *N* is the number of samples, “*l*” stands for a specific diagonal length, while P(l) represents the number of diagonal lines with the specified length “*l*”. The minimum value for $l_{min}$ in (5) and (6) has been set to $l_{min}=2$ [15,18].

To conclude the section, it has to be mentioned that in this article a well-established CRP tool [18] has been used to evaluate the RP, their extension, and the estimators RR, DET, and L.

## 3. Transfer Entropy and Conditional Transfer Entropy

The aforementioned methodology has been compared with the application of the Transfer Entropy (TE) and of the Conditional Transfer Entropy (CTE) [16] on the same set of data.

From the publication of Schreiber [19] onward, the Transfer Entropy has been widely applied in the natural sciences to infer the information flow from a driver to a response system and consequently to infer Granger causality relationships [20].

Considering two physical observables evolving in time, and their representation as two time series “*x*” and “*y*”, using the formalism of the (discrete) Markov process of order “*m*”, it is possible to introduce the TE in a natural way. The underlying assumption is that the probability of the occurrences, for each observable, of their value at the time instance “*n* + 1” depends only on certain number of previously assumed states of the quantity itself. In other words, *x or y* have a memory of order (*l*) or (*k*), respectively. The second assumption is that, if x has a causal influence on y, the past values of *x* allow minimizing the uncertainty in the prediction of *y* at the time “*n +* 1” [16].

Considering the previous assumptions, the TE quantifies the importance of the knowledge $x_{n}$, of a process $x^{\left(k\right)}$, to predict the occurrence of the state $y_{n+1}$ of a process $y^{\left(l\right)}$, also taking into consideration the contribution from the memory of *y* itself. What has just been said can be expressed mathematically in terms of the conditional mutual information between $x^{\left(k\right)}$ and $y^{\left(l\right)}$ or between $x^{\left(k\right)}$, $y^{\left(l\right)}$, and $z^{\left(m\right)}$ when a further quantity is considered as relevant for the information flow [16]:

(8) $$TE_{X\to Y}=I\left(x_{n}^{\left(k\right)},\;y_{n+1}|y_{n}^{\left(l\right)}\right)$$

(9) $$CTE_{X\to Y|Z}=I\left(x_{n}^{\left(k\right)};y_{n+1}|y_{n}^{\left(l\right)},z_{n}^{\left(m\right)}\right)$$

Equation (8) for discrete variables can be written as [19]

(10) $$TE_{X\to Y}=\sum p\left(y_{n+1},x_{n}^{\left(k\right)},y_{n}^{\left(l\right)}\right)\log_{2}\left(\frac{p\left(y_{n+1}|x_{n}^{\left(k\right)},y_{n}^{\left(l\right)}\right)}{p\left(y_{n+1}|y_{n}^{\left(l\right)}\right)}\right)$$

Equation (10) can be easily interpreted also as follows. If the knowledge of $x_{n}^{\left(k\right)}$ does not improve the prediction of $y_{n+1}$, i.e., $p\left(y_{n+1}|x_{n}^{\left(k\right)},y_{n}^{\left(l\right)}\right)=p\left(y_{n+1}|y_{n}^{\left(l\right)}\right)$, then TE = 0. Considering continuous quantities, the definition slightly changes and gets more complicated, but the idea is the same. Similar ideas and assumptions are at the basis of the CTE as well.

It is worth noting in this introduction, that the main issues about the TE lays in the estimation of the entropies in Equations (8) and (9) and consequently in the probability density functions (pdfs). Methods have been established to tackle this issue, like the Kraskov, Stögbauer, and Grassberger (KSG) estimator [21] that extends the Kozachenko–Leonenko estimator [22]. Basically, in the jointly embedded space between $x_{n}^{\left(k\right)},y_{n}^{\left(l\right)}$, a specific norm (usually the max-norm) assesses the distance between kth nearest neighbors and entropies are expressed as functions of this distance and the digamma functions. Further details can be found in literature and are actually out of the scope of the article [16,21].

To conclude the section it has to be mentioned that in this work the well-established JIDT tool presented in [23] has been used to evaluate the TE and the CTE.

## 4. Numerical Tests: Autoregressive Models

The first group of tests has been performed using synthetic data generated with the following family of autoregressive models, constructed on the basis of [24]

(11) $$\begin{array}{c} x_{n}=\left(c_{1}x_{n-1}+c_{2}x_{n-2}+r_{0}\right)+r\left(\mu =0,\sigma =px_{n}\right)\\ z_{n}=\left(c_{3}z_{n-1}+c_{4}z_{n-3}+r_{0}\right)+r\left(\mu =0,\sigma =pz_{n}\right)\\ y_{n}=f\left(x_{n-4}^{\alpha },z_{n-2}^{\beta };c_{5},c_{6},c_{7}\right)+r_{0}+r\left(\mu =0,\sigma =py_{n}\right) \end{array}$$

In (11), $c_{1}=0.95\sqrt{2}$, $c_{2}=-0.64\sqrt{2}$; $c_{3}=0.25\sqrt{2}$ $c_{4}=-0.5\sqrt{2}$; the function $f\left(x_{n-4}^{\alpha },z_{n-2}^{\beta };c_{5},c_{6},c_{7}\right)$ varies depending on the test performed as Table 1 reports. $r_{0}$ stands for a sample drawn from a normal standard Gaussian distribution; $r\left(\mu =0,\sigma =px_{n}\right)$ stands for a sample drawn from a normal Gaussian distribution with σ equals to a percentage “*p*” of the estimated value for $x_{n},y_{n},z_{n}.$ Quantities in (11) have been evaluated using 504 points and discarding the first four samples.

**Figure 1:**
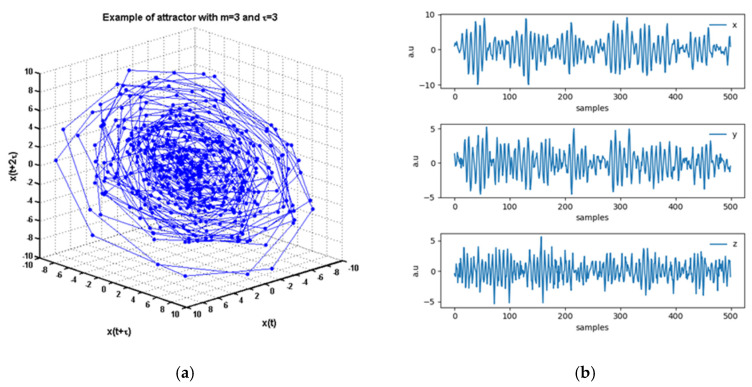
(**a**) Example of attractor with m = 3 and τ = 3; (**b**). $f_{I}^{p=0}\left(\alpha =\beta =1,c_{5}=0.5,c_{6}=0.5\right)$ autoregressive model and the reconstructed delaied embedded shadow attractor with 2.4% of false nearest neighbors [25] built starting from the driver “*x*”.

In (11), both the driver (“*x*”) and the perturbation (“*z*”) influence the response quantity (“*y*”). For each evaluation of Table 1, twelve different realizations of the system (11) have been averaged. Figure 1 provides a picture of the attractor built from *y* for $f_{I}^{p=0}\left(\alpha =\beta =1,c_{5}=0.5,c_{6}=0.5\right)$.

**Figure 2:**
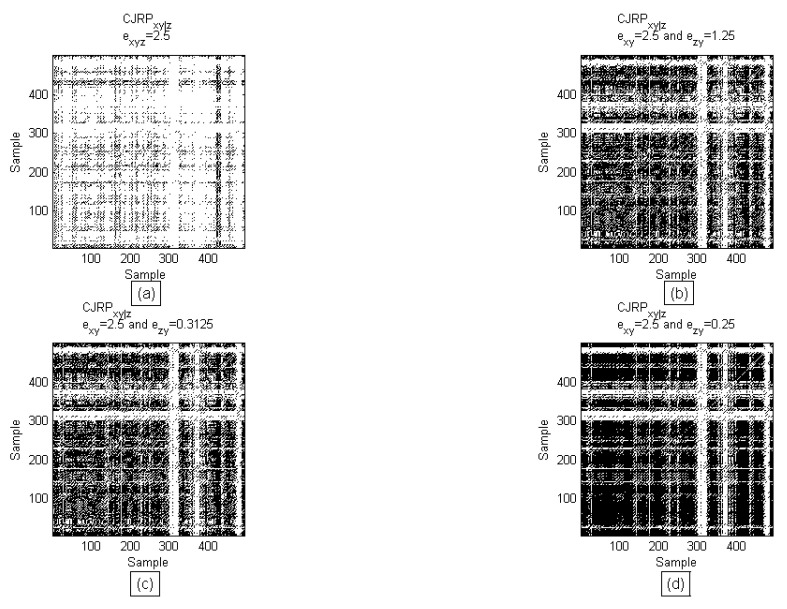
CJRP obtained varying the threshold for the $\epsilon_{JRP_{yz}}$. (**a**) $\epsilon_{JRP_{zy}}=\epsilon_{JRP_{xy}}$; (**b**) $\epsilon_{JRP_{zy}}=\frac{1}{2}\epsilon_{JRP_{xy}}$; (**c**) $\epsilon_{JRP_{zy}}=\frac{1}{8}\epsilon_{JRP_{xy}}$; (**d**) $\epsilon_{JRP_{zy}}=\frac{1}{10}\epsilon_{JRP_{xy}}$ Black pixels stand for occurrences, satisfying the Hadamard product of the definition in Equation (4).

The methodology, based on the estimator described in Equation (4), performs well on autoregressive data. Figure 2 shows the behavior of the CJRP while varying $\epsilon_{JRP_{yz}}$ for the case of the $f_{III}^{p=0}\left(x_{n-4}^{2},z_{n-2}^{2};0.5,\;0.5,0.25\right)$ function. Black pixels indicate positive recurrences of both *x* and *y*.

The most challenging tests have been the ones concerning the nonlinear coupling with or without a correlation term, i.e., $f_{I}^{p=\cdots }\left({}^{\circ}\right)\;\mathrm{and}\;f_{III}^{p=\cdots }\left({}^{\circ}\right)$ with (α,β)=(2,2) according to the notation reported in Table 1, while varying the added percentage of noise *p* and coupling terms $c_{5,6,7}$.

**Figure 3:**
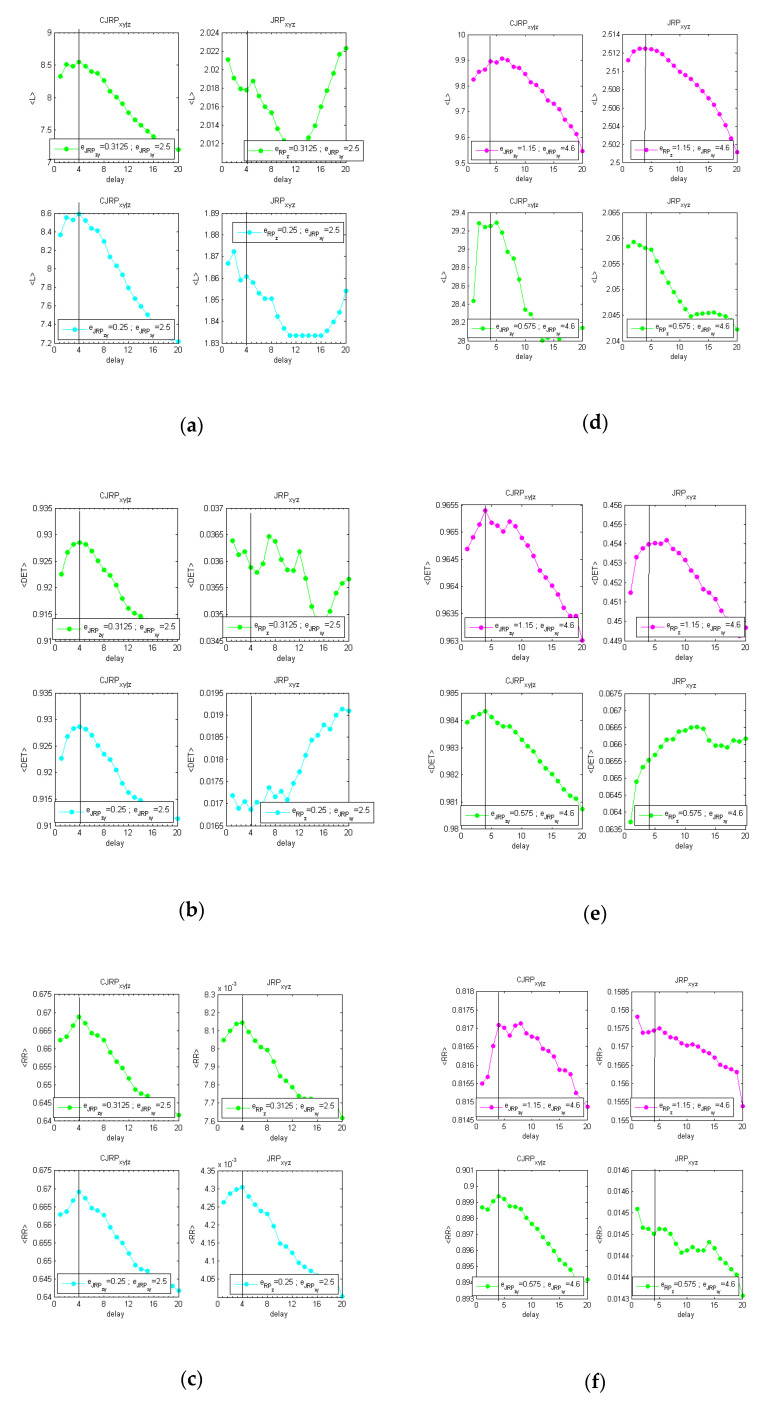
Estimators obtained at two stable stages of the analysis, aimed at determining the actual synchronization lag between *x* and *y*. **Left** [(**a**–**c**), $f_{III}^{p=0}\left(x_{n-4}^{2},z_{n-2}^{2};0.5,\;0.5,0.25\right)$]; **right** [(**d**–**f**); $f_{III}^{p=0.2}\left(x_{n-4}^{2},z_{n-2}^{2};0.5,\;0.5,\;0.25\right)$ ]. (**a**,**d**) The averaged values of L over the replications; (**b**,**e**) the averaged values of the determinism over the 12 replications; (**c**,**f**) the averaged values of RR over the 12 replications. Comparison of the estimators drawn from the CJRP (**left column**) and the JRP (**right column**) varying the $\epsilon_{zy}$ threshold while leaving $\epsilon_{xy}$ one to its optimal value. The overall iterative procedure has been reported in Figure A1 and Figure A2 in Appendix A.

The overall procedure has been reported graphically in Appendix A for one of the difficult cases studied, i.e., $f_{III}^{p=}({}^{\circ}$). Figure 3 reports the behaviors of the average 〈L〉, 〈DET〉, and 〈RR〉, evaluated at two stable stages of the analysis obtained following the methodology described above.

It has also to be stated that the procedure proved to be robust against small errors in the evaluation of the initial guess of the threshold $\epsilon_{\mathrm{xy}}$ [15]. The methodology in fact allowed detecting the right delay between the driver “*x*” and the response system “*y*” even varying the threshold itself over a reasonable interval up to the 30% of its initial estimate.

The embedding dimension has been chosen as the value corresponding to the minimum percentage of false nearest neighbors (FNN). Following the Kennel, Brown, and Abarbanel methodology [25], $R_{tol}=15$, $A_{tol}=2.0$ have been used to evaluate the FNN percentage. At the same time, the optimal lag for the construction of the embedded and delayed space has been set considering the first minimum of the mutual information [25].

Figure 3a–c shows the analysis performed with the same coupling between *x* and *y* ($c_{6}=c_{5}$) and without noise. Figure 3d–f shows the results on the same formulation with 20% of added Gaussian noise.

The estimators suggest in both cases a delay of $d_{xy}=4$. Increasing the noise, the peak at the actual delay appears less distinct from the overall behavior. However, for practical applications to real data, considering a parabolic fit, the indicators show a clear peak at the expected delay.

**Figure 4:**
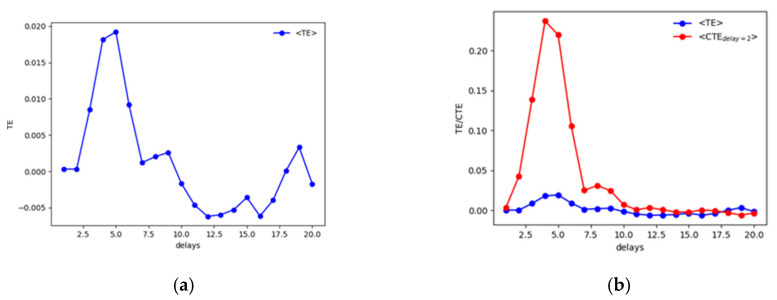
Plot of the averaged Transfer Entropy (TE) (**a**) and Conditional Transfer Entropy (CTE) (**b**) over the replications for the case $f_{III}^{p=0.2}\left(x_{n-4}^{2},z_{n-2}^{2};0.5,\;5,\;2.5\right)$. Negative values of the TE are due to the KSG algorithm used to evaluate the TE itself and implies that the information flow is less than expected and is a symptom of no relationship [16,23].

Results have been compared with the TE and its extension. Figure 4 reports the average value obtained with the use of the TE.

Considering the results of the tests shown in Table 1, it has to be mentioned that the TE cannot always detect the actual delay $d_{xy}=4$, but the use of the CTE clarify the analysis detecting the correct delay at which *z* influences *y*, i.e $d_{zy}=2$.

**Figure 5:**
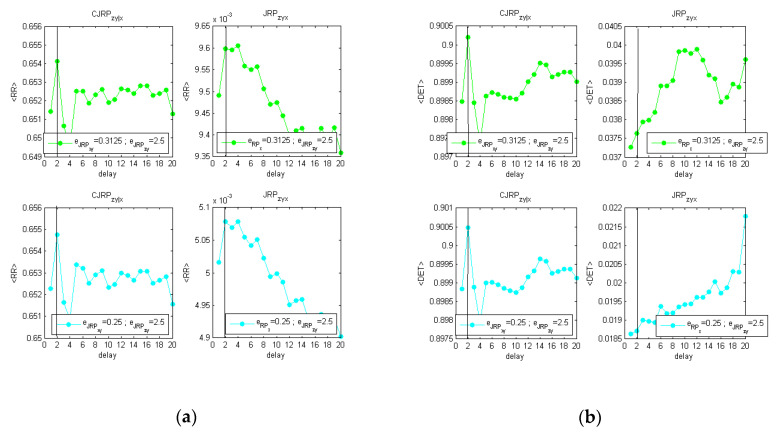
Methodology performed analyzing the lag between *z* and *y* instead of x from y using the CJRP with the following formulations; $f_{III}^{p=0}(x_{n-4}^{2},z_{n-2}^{2};0.5,0.5,0.25$). The actual delay $d_{zy}=2$ emerges in both 〈RR〉 (**a**) and 〈DET〉 (**b**).

To complete the section, another test has been performed to address the capability of CJRP to detect the proper delay between *z* and *y* instead of *x* and *y*. Figure 5 provides the behaviors of the estimators considered, for the case $f_{III}^{p=0}\left(x_{n-4}^{2},z_{n-2}^{2};0.5,0.5,0.25\right)$, stopping the aforementioned methodology once a stable behavior of the CJRP has been reached. Appendix A provides also the plots of the scan performed. As it can be observed, the CJRP can detect the correct delay at $d_{yz}=2$.

## 5. Numerical Tests: Causality Horizon

**Figure 6:**
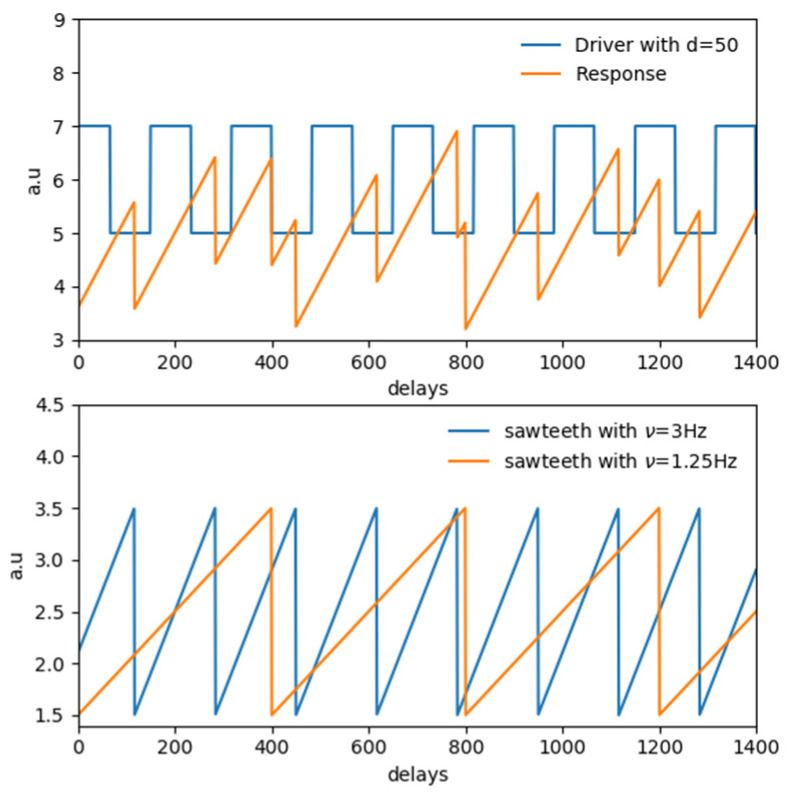
Illustrative example of the second test performed. **Top**: Driver: square function in blue and response observable in orange, due to the sum of the two sawteeth functions used. **Bottom**: sawteeth function considered. In blue the fastest one at 3Hz, delayed of 50 samples with respect to the square function and in orange the slower one at 1.25 Hz.

A second typology of test has been performed, aimed at investigating the causality horizon [26,27,28,29], i.e., the maximum time interval into which two physical quantities are synchronized and in which one observable can be thought as the “drive mechanism” of the second observable, for periodic and quasiperiodic signals of the same nature as those encountered in real experiments in thermonuclear scenarios [30,31]. In this kind of experiment, particular instabilities of the plasma, with potential harmful effects on the machine integrity, are paced by triggering them frequently enough that they do not have time to become dangerous for either the performance or safety of the reactor. The information related both to the evolution of such instabilities and of the occurrence of the pacing, can be retrieved by measuring specific physical quantities in the form of time series. Besides practical implementation difficulties, the evaluation of the pacing efficiency is difficult because the instabilities are quasi periodic, and therefore after a perturbation induced by the control systems, if enough time is allowed to pass, they are bound to reoccur. Typical pacing techniques are the injection of frozen deuterium pellets for controlling ELM instabilities [31] or ICRH power modulation for the control of sawtooth instabilities [30]. The latter experiment has been considered in this paper, by means of a simple synthetic model. A square function with ν=3Hz, 50% duty cycle and two sawteeth functions with different frequencies ($\left[\nu_{1},\nu_{2}]=[3\mathrm{Hz},1.25\mathrm{Hz}\right]$) have been considered. A delay of 50 samples has been added to the fastest sawteeth function with respect to the square wave, then the two have been summed as shown in Figure 6. The resulting time series are meant to simulate the modulation of a fast (paced) sawtooth by a square wave modulation in presence of a slower (natural) sawtooth behavior [30,31].

For this test, 1500 samples have been generated and the first 100 removed. Normal Gaussian noise with a standard deviation equals to p=[0.01, 0.05,0.1] of the values assumed by the periodic functions has been also added on data.

With regard to the nomenclature, the square function, the driver, has been referred to as “*x*”, the response, i.e., the fastest and delayed sawteeth as “*y*”, i.e., the slower sawteeth function and the external perturbation, as “*z*”.

**Figure 7:**
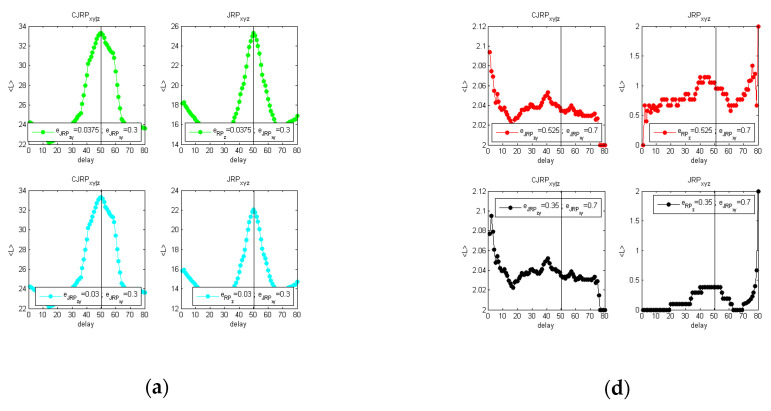

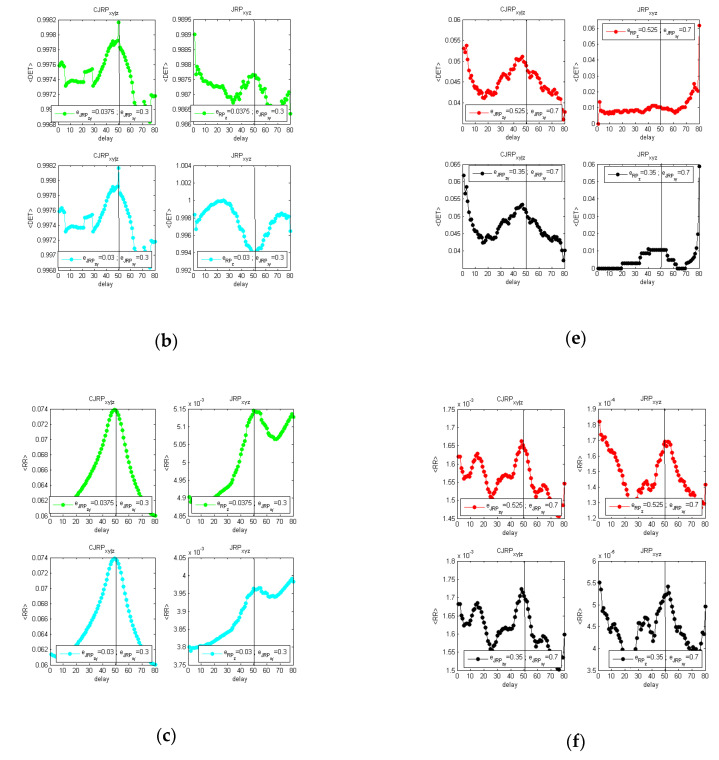
Methodology performed to remove the influence of z from y in the occurrence analysis using the CRJP for the case of Figure 6. **Left**: (**a**–**c**) without noise; **right**: (**d**–**f**) adding a 10% of noise on all the time series considered. In panels (**a**,**d**) L; in panels (**b**,**e**) Determinism; in panels (**c**,**f**) RR. Comparison of the CJRP (**left column**) and JRP estimators (**right column**) while varying the $\epsilon_{zy}$ threshold while leaving the $\epsilon_{xy}$ at its optimal initial value.

Results are reported in Figure 7. All the estimators for the noiseless test show the correct delay at $d_{xy}=50$. Adding the noise, from the results in Figure 7d–f, it can be seen how the L estimator is not very informative in this case, while the correct delay can be detected and more clearly observed using DET and RR.

**Figure 8:**
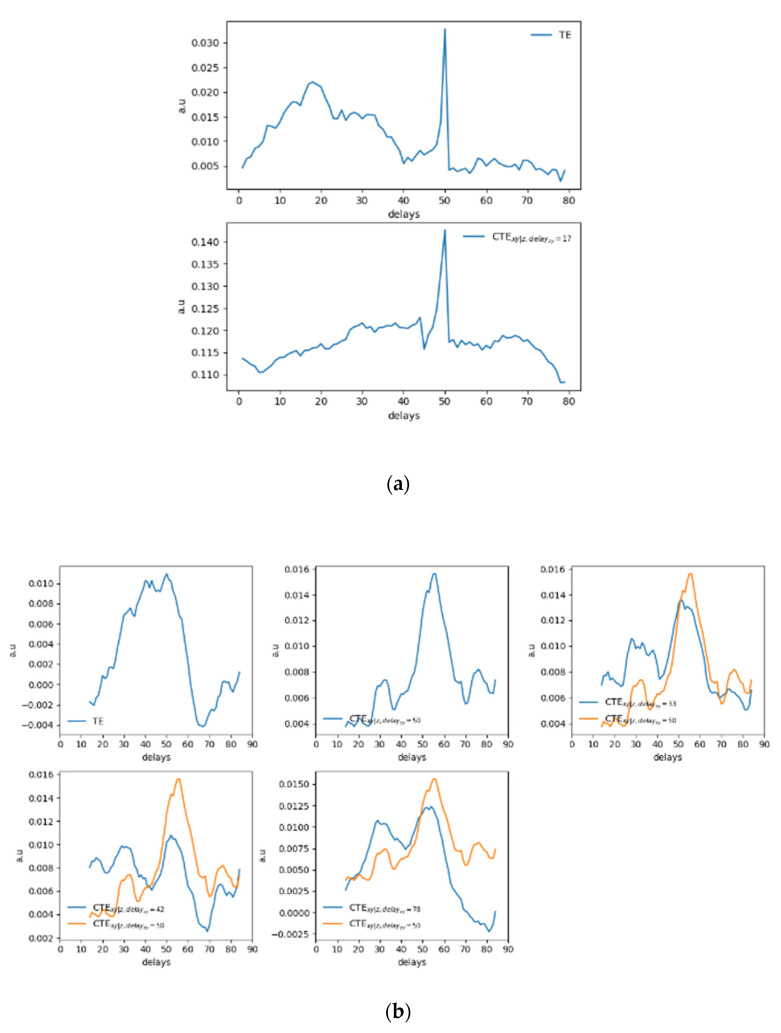
Behaviors for the synthetic signals analyzed and shown in Figure 6 with (**a**) 0% of noise and (**b**) 10% of Gaussian noise on data. Negative values of the TE are due to the KSG algorithm used to evaluate the TE itself and implies that the information flow is less than expected and is a symptom of no relationship [16,23].

Again, results are compared with the use of the TE and the CTE. As it can be observed in Figure 8a for the noise-free case or in Figure 8b for the 10% of added noise.

Considering Figure 8a, the TE allows estimating the correct delay between the driver and the response system, but spurious peaks appear in both Figure 8a,b. In real applications, the actual delay of $d_{xy}=50$ samples would not be detected directly. The CTE improves the results especially for the noiseless data in Figure 8a. However, the behavior of the CTE with noised data (Figure 8b) requires a deeper and nontrivial analysis that might lead to wrong assessments when analyzing real data with periodic or quasiperiodic behaviors, typical of synchronization experiments in magnetically controlled nuclear fusion. Similar to the previous tests, it has to be considered that the CTE depends on two delays: one between the driver “*x*” and response “*y*” ($d_{xy}$) and another between the secondary driver, i.e., the perturbation, “*z*” and the response quantity itself ($d_{zy})$. Fixing a certain value for $d_{zy}$, a behavior of the CTE as a function of $d_{xy}$ can therefore be observed. Scanning the possible delays between the two sawteeth functions (“*z*” and “*y*”), i.e., changing the $d_{zy}$ values and observing the behavior of the CTE, completely different behaviors emerge in which the maximum of the CTE at a specific $d_{xy}$ cannot be detected clearly. This is basically due to the periodic characteristic of the signals and of the secondary driver. Consequently, the delays $d_{zy}$ to set in order to proceed with the analysis, i.e., in order to detect the $d_{xy}$ at which the maximal influence of “*x*” on “*y*” can be addressed, have been selected considering the local maxima observed from the ${\mathrm{TE}}_{x\to y}$. In such a way, it has been noticed that the local peak at $d_{xy}=50$ remained almost unaltered as it can be observed in Figure 8b, being the actual delay between the driver and the response system and consequently the maximum causality horizon for the signals analyzed.

On the other hand, the analysis, performed with the CJRP and shown in Figure 7, is actually less vulnerable to possible misunderstanding. Secondary peaks appear, but the actual one at the expected delay is identified directly by two estimators out of the three considered without any other further interventions.

## 6. Extension to More than Three Observables

**Figure 9:**
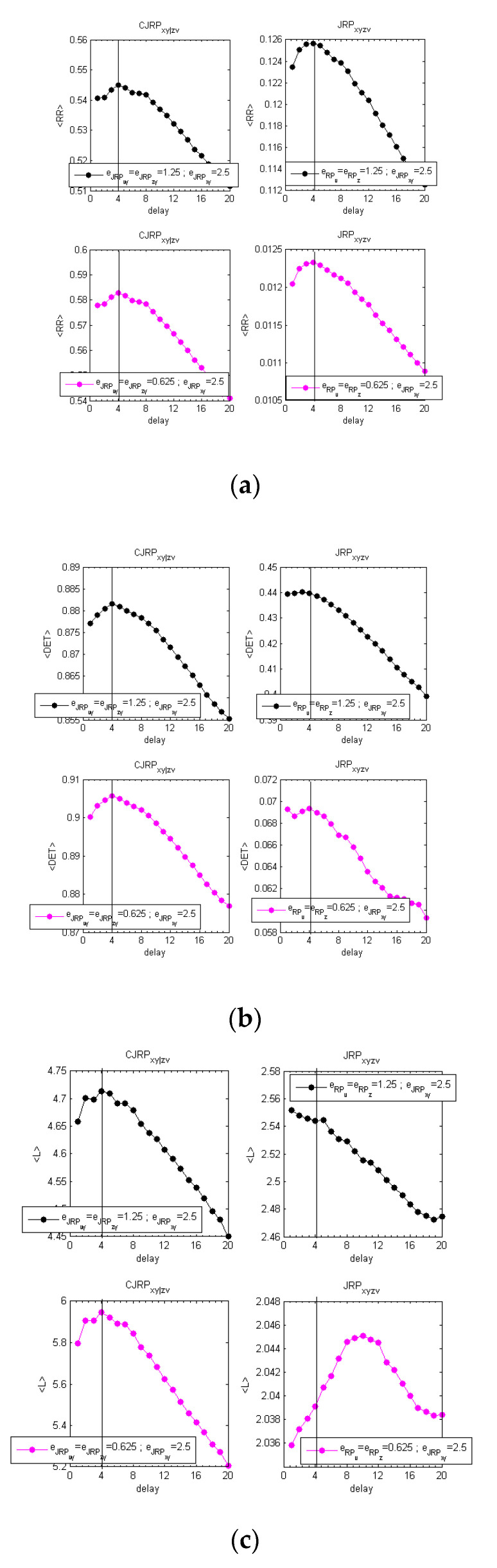
Behaviors of <RR> (**a**), <DET> (**b**), and <L> (**c**), and for the system in (12). The study is aimed at highlighting the synchronization between *x* and *y*, minimizing the effect of both *z* and *v* from *y*. Considering (12), the expected delay is $d_{xy}=4$.

In all natural sciences it is not uncommon to deal with multiple sources affecting the same observable, i.e., the response system. An important example in nuclear fusion deals with the need of avoiding disruptions [32,33]. Disruptions are abrupt and unwanted events occurring in magnetically controlled nuclear fusion tokamaks due to the rapid loss of the plasma confinement, usually in a time scale of few milliseconds, causing the termination of the pulse and having the potential to cause serious damages to the reactor. While it is known nowadays that many factors concur to the occurrence of this kind of events, neither their relative importance nor the sequence of the triggering factors are fully understood. Consequently, to investigate the possibility of using the CJRP with more than three variables, tests have been conducted and an example is reported in this section. Another observable “*v*” has been introduced in (11) and coupled linearly with *y* with a delay of $d_{vy}=6$. The system used to generate the synthetic data is reported in Equation (12), that actually extends (11) and has also been built on the basis of the work in [24],

(12) $$\begin{array}{c} x_{n}=0.95\sqrt{2}x_{n-1}-0.64\sqrt{2}x_{n-2}+r_{0}\\ z_{n}=0.25\sqrt{2}z_{n-1}-0.5\sqrt{2}z_{n-3}+r_{0}\\ v_{n}=0.85\sqrt{2}v_{n-2}-0.35\sqrt{2}v_{n-4}+r_{0}\\ y_{n}=0.5x_{n-4}+0.5z_{n-2}+0.5v_{n-6} \end{array}$$

where $r_{0}$ stands for a sample drawn from a normal standard Gaussian distribution. For this analysis, 506 samples have been evaluated and the first six were discarded. The objective of this test is to confirm that the approach of the CJRP can identify the proper delay between a driver and a target in presence of more than one disturbing influence. The methodology described in the previous sections has been applied and the results reported here for the stable region, again using twelve repetitions of the system in (12). In this example, the threshold $\epsilon_{zy}$ and $\epsilon_{vy}$ have been reduced iteratively. The CJRP allows estimating quite easily the correct synchronization between *x* and *y* at the delay $d_{xy}=4$ as can be derived by simple inspection of Figure 9.

## 7. Presence of Outliers

**Figure 10:**
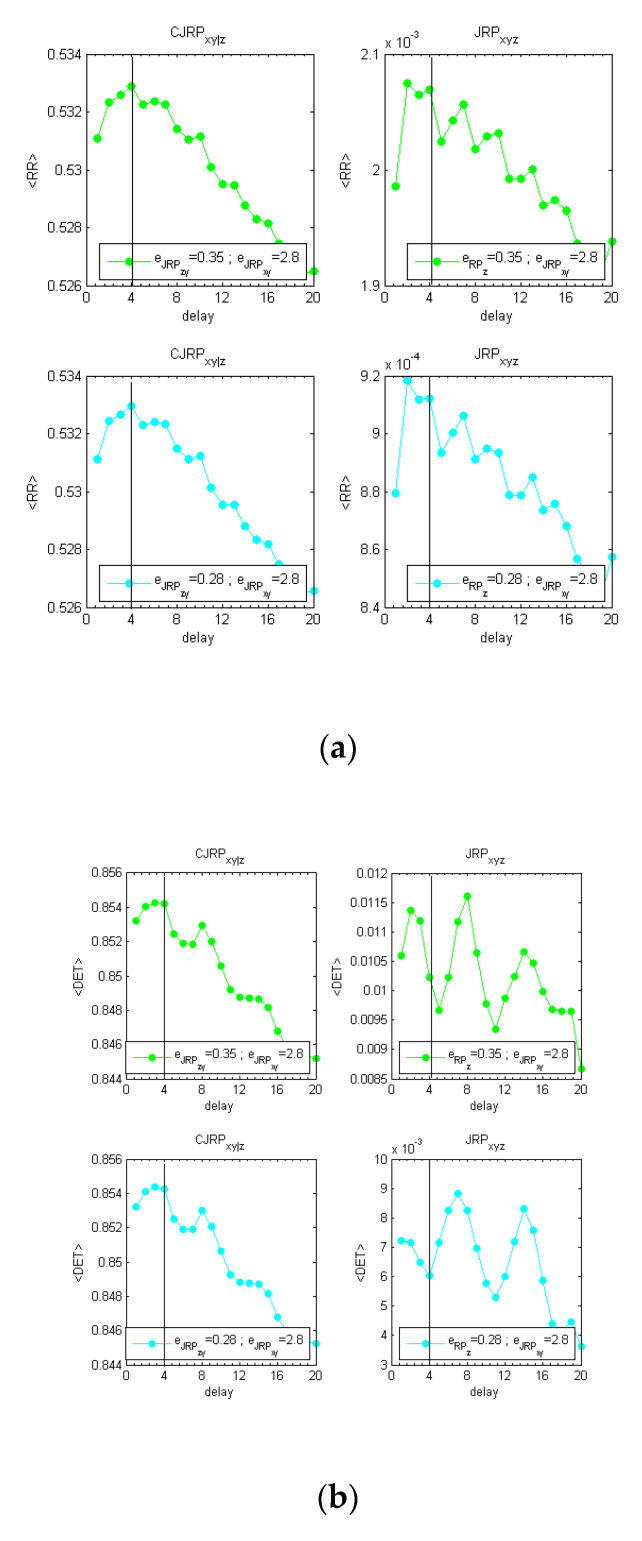
Behaviors of <RR> and <DET> in panels (**a**,**b**), respectively, for the case $f_{III}^{p=0.1}\left(x_{n-4}^{2},z_{n-2}^{2};0.5,\;5,\;2.5\right)$ considering outliers in data. The methodology detects the correct delay at $d_{xy}=4$, with the exception of the <L> estimator that has not been reported.

**Figure 11:**
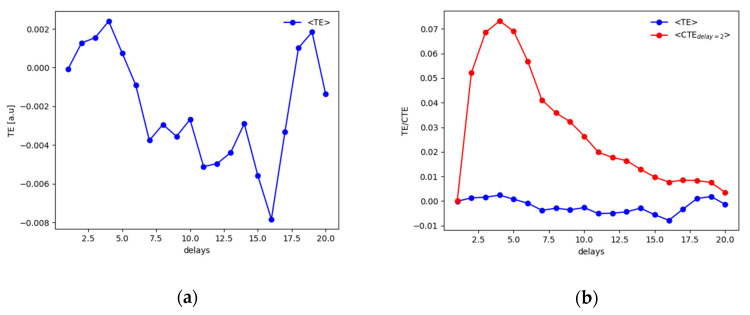
Behavior of (**a**) TE and (**b**) CTE for the autoregressive system in presence of outliers as described in Equation (13). The TE detects the delay at $d_{xy}=4$ and a spurious one at $d_{xy}=19$ with almost the same amplitude, while the CTE finds the actual delay $d_{xy}=4$ at $d_{zy}=2$.

As a final test, also the presence of outliers has been investigated. Here, the results obtained for the $f_{III}^{p=0.1}\left(x_{n-4}^{2},z_{n-2}^{2};0.5,\;5,\;2.5\right)$ relationship described in Table 1 have been reported as the formulation belongs to one of the most challenging cases studied. The test has been performed considering the system described in (11), but adding for each element $x_{n},y_{n},z_{n}$ a Gaussian randomly picked value, i.e., $N_{x_{n},y_{n},z_{n}}\left(\mu ,\sigma \right)$, according to the following rule,

(13) $$N_{x_{n},y_{n},z_{n}}\left(\mu ,\sigma \right)=\{\begin{array}{c} N\left(\mu =0,\sigma =p\left(x_{n},y_{n},z_{n}\right)\right)\;if\;g\in U\left[0,1\right]<0.2\\ N\left(\mu =\left(x_{n},y_{n},z_{n}\right),\sigma =p\left(x_{n},y_{n},z_{n}\right)\right)\;if\;g\in U\left[0,1\right]\geq 0.2 \end{array}$$

where U[0,1] indicates a uniform distribution between 0 and 1 from which a scalar g value is picked. Results are reported in Figure 10 for the two stable iterations of the methodology suggested and in Figure 11 for the TE and CTE. RR and DET identify correctly the actual delay between *x* and *y*. On the other hand, in this case it can be observed how the TE cannot evaluate clearly the correct delay as another spurious peak $d_{xy}=19$ emerges of almost the same amplitude as the right one; the CTE on the contrary provides the appropriate information.

To complete the section, another test with different percentages of outliers (10%, 20%, or 30%) has been reported. In this case, either a Normal or a Skew-normal distribution has been used as pdf to generate the outliers themselves. For coherence, results regarding the same relationship $f_{III}^{p=0.1}\left(x_{n-4}^{2},z_{n-2}^{2};0.5,\;5,\;2.5\right)$ reported previously have been used. The time occurrence of an outlier has been chosen randomly, then considering the Normal case, it has been drawn from $N\left(\mu =\left(x_{n},y_{n},z_{n}\right),\sigma =0.1\left(x_{n},y_{n},z_{n}\right)\right)$. For the Skew-normal [34] case instead, for each outlier, a scalar “g” has been picked up first from a uniform distribution, then if g∈U[0,1]≤0.5 the skewness parameter has been set to “4”, otherwise to “−4”. Results are coherent in both cases with the test reported in the previous paragraph. Already with 10% of outliers, indeed the TE cannot detect the expected delay and an analysis based on this estimator would suggest a weak or no correlation between the driver and the response quantity. Considering the CTE instead, behaviors are similar to Figure 11b. Setting the right delay of $d_{zy}=2$ between the quantity “*z*” and the response one, the maximum of the curve can be observed at either $d_{xy}=3$ or $d_{xy}=4$, without a sensible difference between the two values. Increasing the number of outliers, a lower value of the maximum detected by the CTE is observed. While the ${\mathrm{JRP}}_{\mathrm{xyz}}$ does not detect the correct delay between the driver and the response quantity, considering the CJRP instead, as in the previously reported test the “L” estimator is not informative, but the RR and the DET detect correctly a delay between the driver and the response system at either $d_{zy}=4$ or $d_{xy}=5$. The higher the number of outliers, the more oscillations are observed. Similarly with the CTE, not a particular dependence has been observed on the type of pdf used.

## 8. Discussions on Lines of Future Investigations

In terms of future applications in thermonuclear fusion, the developed tools could contribute to the development of integrated scenarios for ITER [35] and to the refinement of diagnostics for the magnetic fields [36,37]. Other interesting challenges would be the determination of causal relations between various atmospheric phenomena in the environmental sciences, whose periodic nature renders detrending and interpretation very delicate [38].

## 9. Conclusions

In this work, a new type of Recurrence Plot has been defined: the Conditional Joint Recurrence plot or CJRP. Its properties have been tested using synthetic data generated with autoregressive linear, nonlinear noised, and noise-free models, with different couplings, using also periodic noised and noise free synthetic signals and including outliers. Results have shown the capability of the CJRP to identify the actual time delay of the causal influences, removing the effects of external perturbations up to a relevant level of noise added to the data. The outcomes of the tests have been averaged over twelve repetitions to provide a significant statistical basis to the conclusions. It should be mentioned that, in terms of individual realizations, 92% of correctly detected cases has been observed in general. In particular, the lowest scoring has been obtained for the nonlinear case $f_{III}^{p=0.2}\left(x_{n-4}^{2},z_{n-2}^{2};0.5,\;0.5,\;0.25\right)$ with a 75% of success rate. This is thought to be due to the typology of the formulation analyzed and to the specific noise added.

Different formulations have been tested before selecting the one in (4), also using the Cross Recurrence Plots. However, the best results have been obtained with the formulation reported.

The great flexibility of the Joint Recurrence Plots, to include more quantities, simply adding terms in the Hadamard product, allows the CJRP to be easily extended to more than three quantities. Tests have been performed using the autoregressive system studied in this article and the results confirm what was just stated.

In conclusion, for practical purposes, the proposed CJRP can be implemented to complement the transfer entropy and the conditional transfer entropy. On the other hand, the CJRP approach presents various advantages: it is both simpler to implement and interpret and can also be extended immediately to higher numbers of variables, which presents a very serious challenge for TE and CTE.

## Figures and Tables

**Table 1:** Definition of the functions analyzed for the autoregressive test.

Type	f()	Parameters
$f_{I}^{p=\cdots }\left({}^{\circ}\right)$	$c_{5}x_{n-4}^{\alpha }+c_{6}z_{n-2}^{\beta }$	$c_{5}=0.5,c_{6}=\left[c_{5},2c_{5},5c_{5},10c_{5}\right]$ (α,β)=[(1,1),(1,2),(2,1),(2,2)] p=[0, 0.1, 0.2]
$f_{II}^{p=\cdots }\left({}^{\circ}\right)$	$c_{7}x_{n-4}\cdot z_{n-2}$	$c_{7}=\left[0.25,0.5,1.25,2,5\right]\;p=\left[0,\;0.1,\;0.2\right]$
$f_{III}^{p=\cdots }\left({}^{\circ}\right)$	$c_{5}x_{n-4}^{\alpha }+c_{6}z_{n-2}^{\beta }+c_{7}x_{n-4}\cdot z_{n-2}$	$c_{5}=0.5$ $(c_{6},c_{7})=\left[\left(c_{5},0.25\right),\left(2c_{5},0.5\right),\left(5c_{5},1.25\right),\left(10c_{5},5\right)\right]$ (α,β)=[(1,1),(1,2),(2,1),(2,2)] p=[0,0.1,0.2]

**Figure A1:**
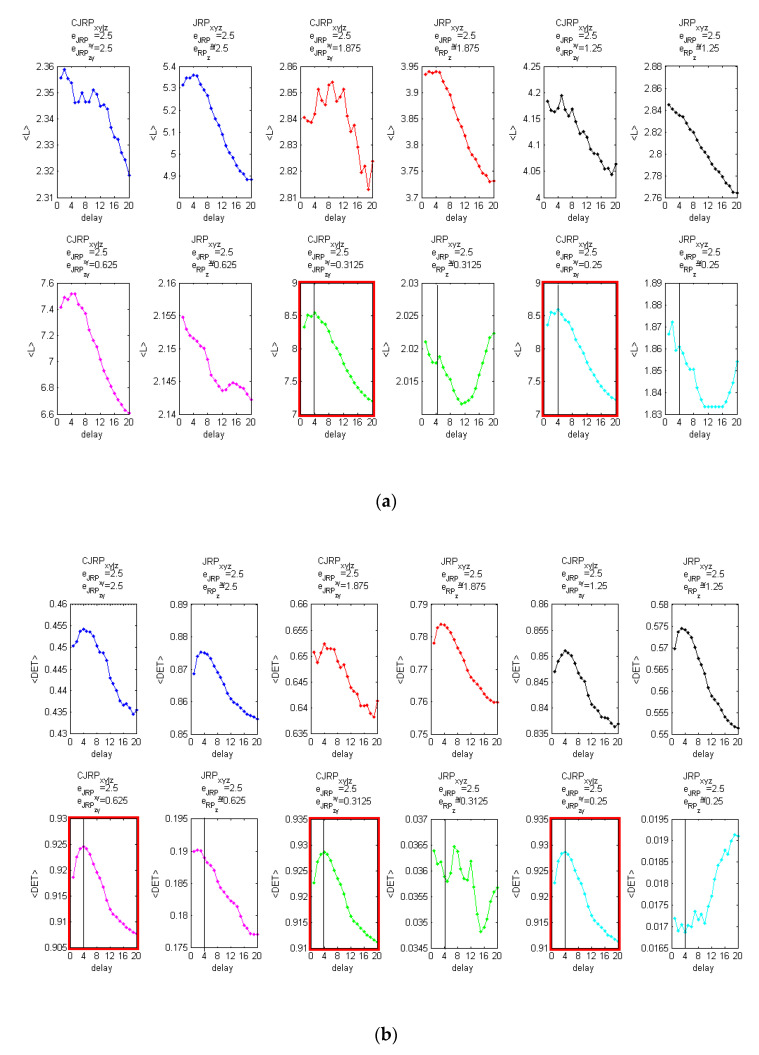

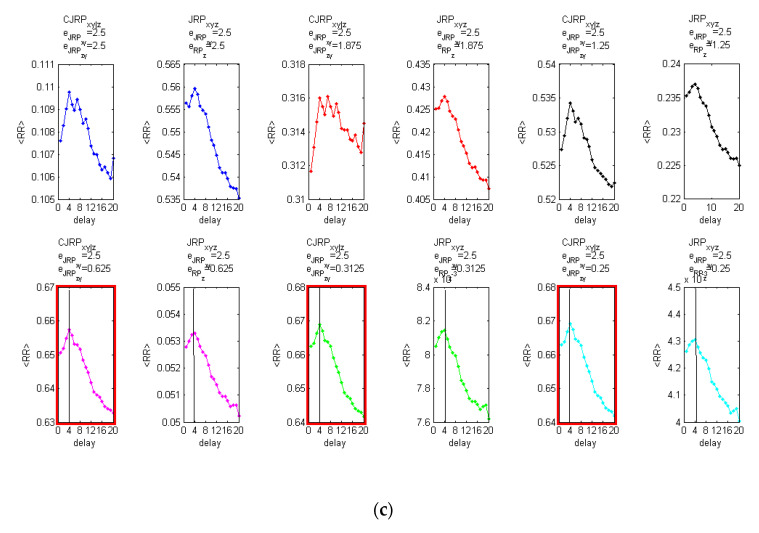
Behaviors of <L> (**a**), <DET> (**b**), and <RR> (**c**) for $f_{III}^{p=0.0}\left(x_{n-4}^{2},z_{n-2}^{2};0.5,0.5,0.25\right)$. The study is aimed at inferring the correct delay between *x* and *y* at $d_{xy}=4$, while minimizing the influence of z. Vertical black lines have been added at the expected delay between the driver and the response system. The most informative plots have been put in a red and ticker frame.

**Figure A2:**
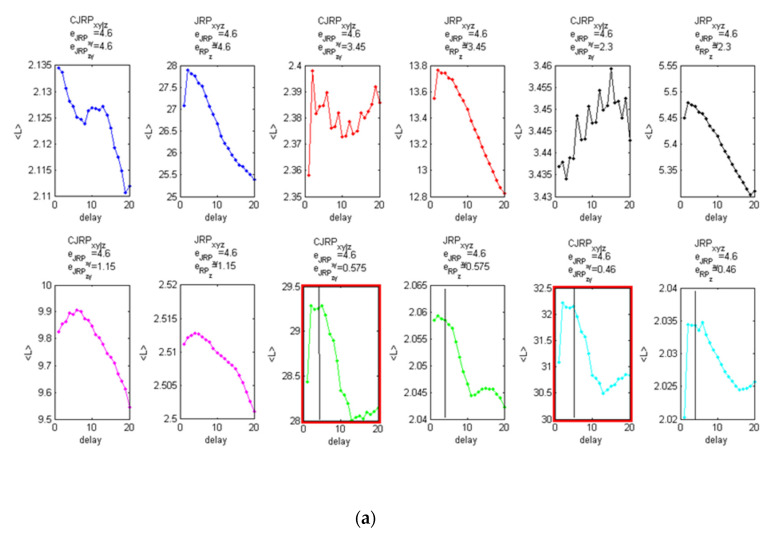

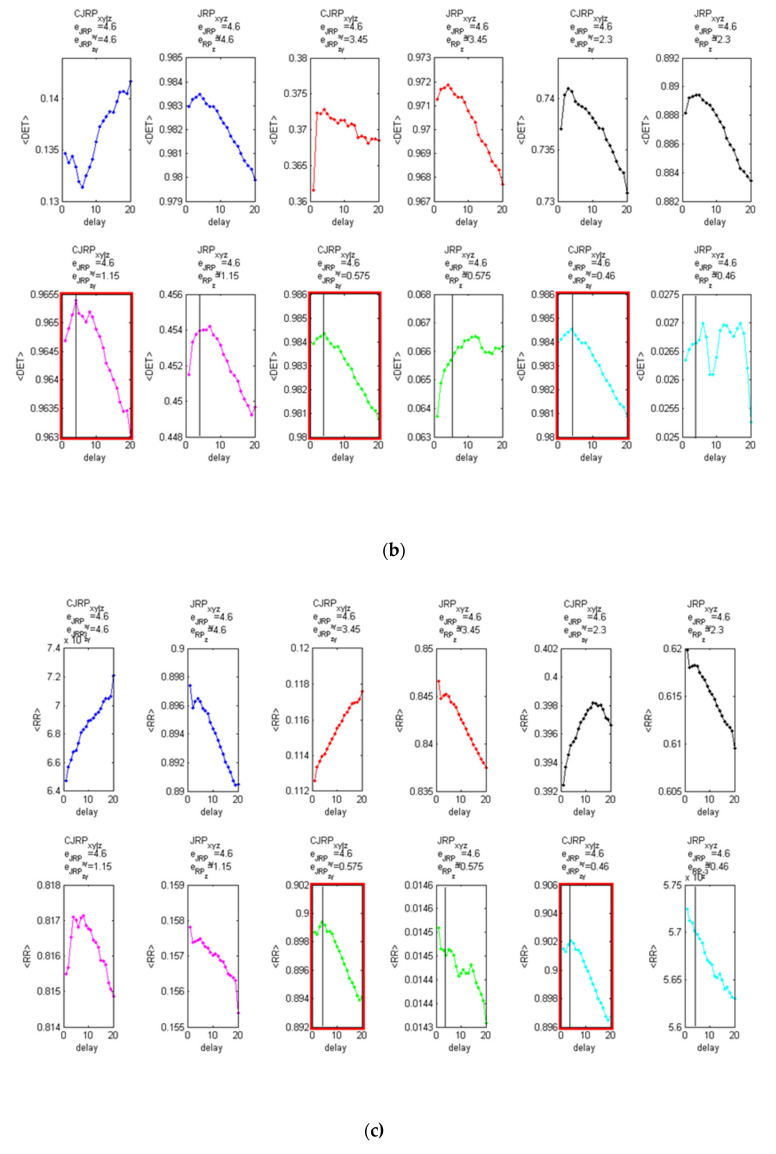
Behaviors of <L> (**a**), <DET> (**b**), and <RR> (**c**) for $f_{III}^{p=0.2}\left(x_{n-4}^{2},z_{n-2}^{2};0.5,0.5,0.25\right)$. The study is aimed at inferring the correct delay between *x* and *y* at $d_{xy}=4$, while minimizing the influence of z. Vertical black lines have been added at the expected delay between the driver and the response system. The most informative plots have been put in a red and ticker frame.

**Figure A3:**
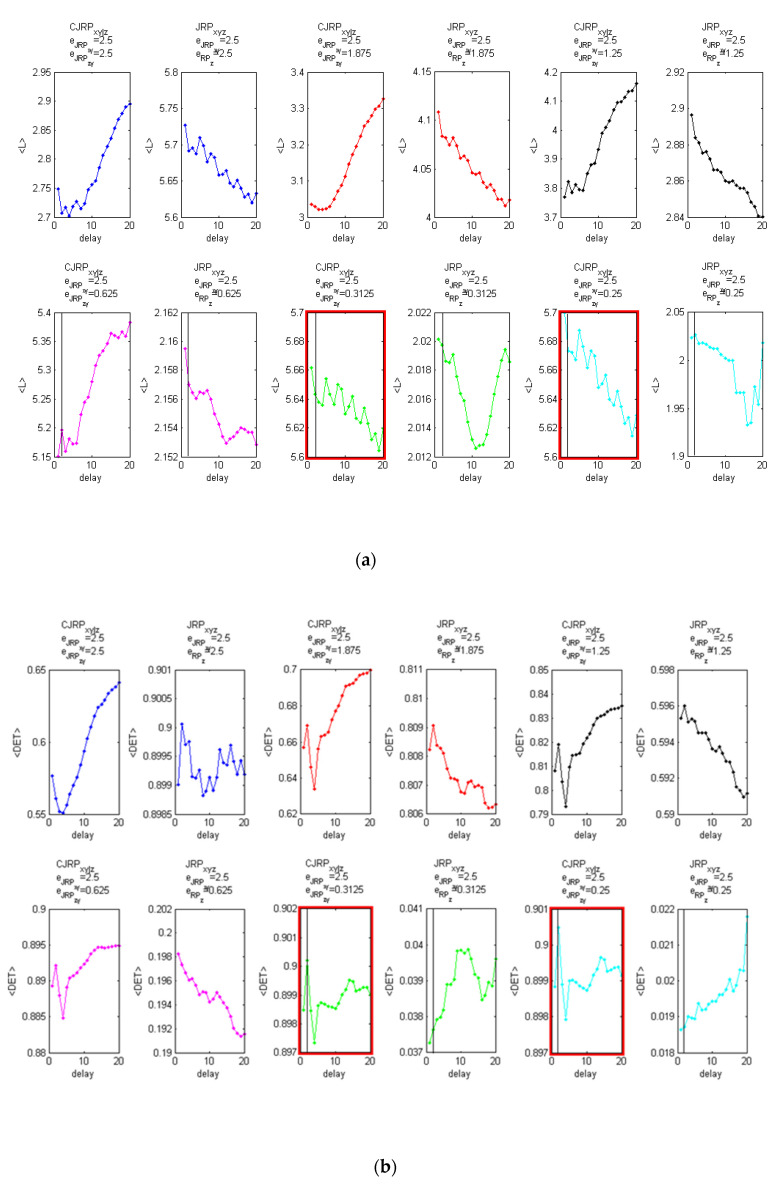

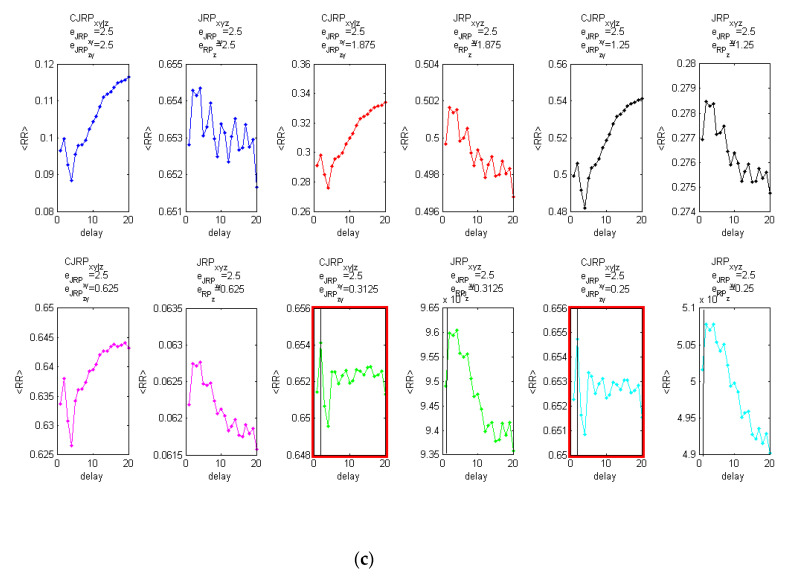
Behaviors of <L> (**a**), <DET> (**b**), and <RR> (**c**) for $f_{III}^{p=0.0}\left(x_{n-4}^{2},z_{n-2}^{2};0.5,0.5,0.25\right)$. The study is aimed at inferring the correct delay between *z* and *y* at $d_{zy}=2$, while minimizing the influence of *x.* Vertical black lines have been added at the expected delay between the driver and the response system. The most informative plots have been put in a red and ticker frame.

## Appendix A

In this appendix, the plots regarding the methodology described in the article have been reported. The iterative procedure via the estimators used to quantify the CJRP can be observed. Figure A1 and Figure A2 are related to the study reported in Figure 3, while Figure A3 is related to the counter example reported in Figure 5.
